# Evaluating the Impact of Information System Quality on Continuance Intention Toward Cloud Financial Information System

**DOI:** 10.3389/fpsyg.2021.713353

**Published:** 2021-08-19

**Authors:** Yanxing Li, Jinghai Wang

**Affiliations:** Planning and Finance Department, Gansu Agricultural University, Lanzhou, China

**Keywords:** relationship quality, cloud financial information system, information quality, system quality, service quality, partial least squares

## Abstract

Cloud financial accounting informatization is a product of the combination of accounting work and information technology, a current demand for financial information management in the new era, and a necessary means for enterprises to enhance their core competitiveness. Therefore, this study is based on DeLone and McLean's information system success model, and through theoretical interpretation and derivation, we integrate satisfaction and trust in relationship quality to measure the impact of users' intention to continue using the cloud financial accounting system. A sample of 289 faculty and finance staff with experience in using cloud financial accounting systems was used to test the hypotheses using Partial Least Squares (PLS). The results of the study showed that (1) user participation had a significant positive impact on satisfaction with the system quality, information quality, and service quality of the cloud financial accounting system; (2) the system quality and service quality of the cloud financial accounting system had a significant positive effect on user trust; and (3) the quality of the cloud financial accounting system had a mediating effect on intention to continue using the system through satisfaction and trust. Since there is a lack of research on the antecedents and outcomes of user linkage to cloud financial accounting systems in the literature, especially the empirical results on the mediation relationship from the perspective of relationship quality. Therefore, this study can fill above mentioned research gap and provide specific recommendations for sustainable management practice.

## Introduction

Due to the rapid development of information technology, the transmission and management of information has become more complex and time-sensitive, and enterprises and government departments have been implementing various information management systems to enhance operational efficiency and organizational effectiveness (Banker and Kauffman, 2004). Cloud financial accounting informatization is a product of the combination of accounting work and information technology, and it is also a current demand for financial information management in the new era, and a necessary means for enterprises to enhance their core competitiveness (Wang et al., 2019). With the gradual popularization of big data technology, on the one hand, it provides new development opportunities for cloud financial accounting informatization, and on the other hand, it also brings information security issues. Enterprises need to sustainably explore the potential risk prevention for cloud financial ac-counting informatization in the era of big data to promote the stable and comprehensive development of enterprise financial accounting informatization (Wang et al., 2019).

In the current context of information technology, many companies sign sales contracts with customers through computer networks, thus forming digital sales contracts. The finance staff can verify the authenticity of the customer's identity by examining the validity and legality of the contract. Unlike traditional paper-based contracts, electronic contracts are digitally stored in such a way that various documents and books of accounts cannot be directly identified by the human eye and need to be examined with the help of a computer (Dai and Vasarhelyi, 2017; Song et al., 2018). In addition, when finance staff query the results of business processing, sometimes they can only query the results of business processing, and it is difficult to find the source of the business, and the inability to determine the reliability of the source of the business will increase the risk of cloud financial and accounting information work. In the cloud financial accounting information work, if the financial staff modify the financial information due to operational errors, it is often difficult to verify.

The financial accounting system to the cloud version, its conversion process is bound to cause users in the use of the positive and negative views, mainly because the original system if the stand-alone version, to the cloud version can be operated in places where there is a network, you can work at home, however, the cloud version is not necessarily more stable than the stand-alone version, and must have a network to operate, there is the problem of network connection speed, and applicable to more objects once the hardware and software systems must be strengthened, the investment costs are bound to be considerable. Costly failures on the implementations of information systems continued to be reported in the prior empirical studies (Bourne, 2005; Gargeya and Brady, 2005). In the context of changing and updating information system requirements, organization members must have positive feelings about the information quality, system quality, and service quality features provided by the information system in order to be willing to use it continuously (Roky and Al Meriouh, 2015; Hariguna et al., 2017; Aldholay et al., 2018; Verma et al., 2018; Jeyaraj, 2020). Therefore, it is important to study how to make the cloud financial accounting system work successfully, enhance the satisfaction and trust of members and bring overall benefits to the organization.

With the advancement of technology, the service industry has gradually shifted from a service type that requires a high degree of interpersonal contact in the early days to a service type with a lot of technological intervention, and the type of service contact has also changed. Early research on service encounters focused on the dynamics and characteristics of service worker-customer interactions; in recent years, prior studies have actively explored the important role of technology in service encounters (Bitner et al., 2000; Dabholkar and Bagozzi, 2002; Chen et al., 2009; Lin et al., 2011). Although technology involved services have become common, it does not mean that technology access is widely accepted by the public. Therefore, the continued adoption of cloud financial accounting systems by users can be considered a fundamental issue for the success of the platform, especially in today's competitive enterprise resource planning industry, where users can quickly change their preferred service provider in a cost-effective manner. In addition, since cloud financial accounting systems not only require continuous use by users, but also rely on their trust in the information provided by the system.

In the current business context, enterprises are increasingly to implement information systems to fulfill long-term competitive advantage, performance growth, and opportunities to ensure sustainable success (Ghobakhloo and Tang, 2015; Al-Okaily et al., 2020). Implementing information systems into enterprises can furnish extensive benefits, especially for developing countries (Solaymani et al., 2012). In effect, some recent studies have shown that enterprises in developing countries are investing substantially in information systems to sustain their competitive position (Tang and Ghobakhloo, 2013; Ghobakhloo and Tang, 2014). Reviewing the past literature, the information systems success model (DeLone and McLean, 2003) provided the direction of academic and practical research in information systems success performance measurement and causality model to explain the impact and evaluation of information system quality on user satisfaction and trust (Roky and Al Meriouh, 2015). Therefore, this study adopts the information system success model from DeLone and McLean's (2003) to investigate the technical (information quality, system quality, and service quality) and relevant factors (satisfaction, trust, and continuance intention) as the main axes of the study.

## Theoretical Background and Literature Review

### Information System Quality

DeLone and McLean (1992) proposed six components to evaluate a successful information system: system quality, information quality, use, user satisfaction, individual im-pact, and organizational impact. To evaluate the success of information system operations, this evaluation model should include the quality characteristics of the information system itself (i.e., system quality), the quality of the information system output (i.e., information quality), the consumption of the information system output (i.e., use), the response of in-formation system users to the information system (i.e., user satisfaction), the impact of the information system/information technology on user behavior (i.e., individual impact), and the impact of the information system on organizational performance (i.e., organizational impact). performance (i.e., organizational impact). System quality and information quality influence positively the users' usage and satisfaction of the information system, and the users' usage and satisfaction affect each other and then affect their personal behavior, which in turn affects the performance of the organization.

**Figure 1:**
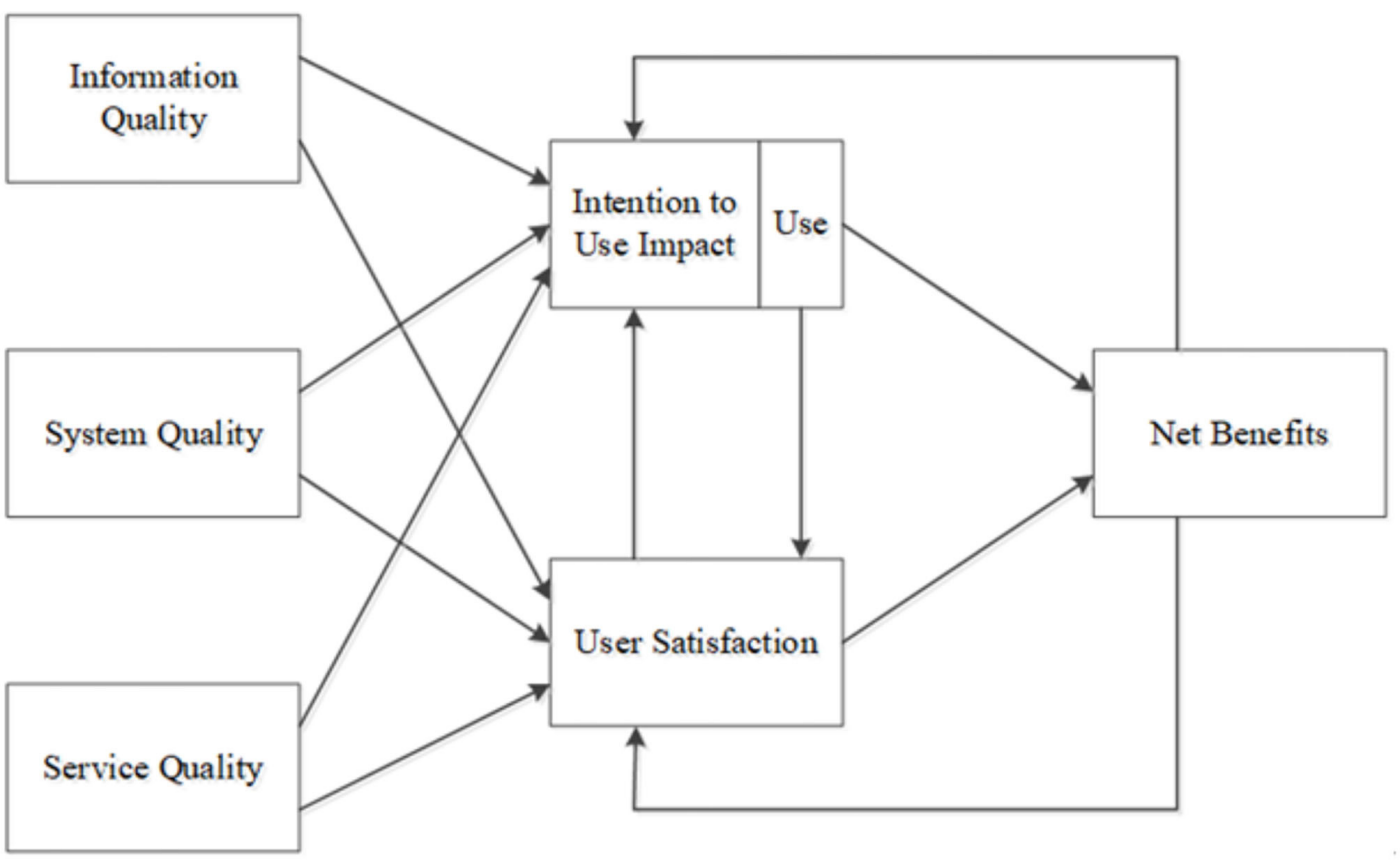
Information system success model (DeLone and McLean, 2003).

After DeLone and McLean (1992) developed Information Systems Success Model, many scholars have adopted this model to explore the issue of organization-led information systems (Seddon and Kiew, 1996; Myers et al., 1997; Seddon, 1997; Kulkarni et al., 2006). Pitt et al. (Pitt et al., 1995) conducted a survey on service quality of information systems and found that changing the attitude of information department personnel, changing the attitude of information system users, setting expectations of information personnel, improving communication with customers, and improving information system services (i.e., customer- and user-oriented) would improve the service quality of information systems. Service Quality and Continuity of Use: The importance of service quality in information systems has also been identified in empirical studies by several scholars (Kettinger and Smith, 2009). In 2003, DeLone and McLean supplied the service quality as a component and modified the model to include system quality, information quality, and service quality as dimensions affecting the user satisfaction and successful usage of the information system, as subsequent indicators to illustrate the success of the information system from a cause-and-effect perspective. System benefits are also assessed by the feedback relationship between system usage, user satisfaction and system benefits, and are considered as indicators of the success framework of the information system (as shown in Figure 1).

When users are satisfied with the system quality and information quality of the in-formation system, it is beneficial to individual performance, and through the use of the system, it can promote the efficiency and ability of work, and individual efficiency improvement leads to enhance organizational development. Most of the previous studies examined the effectiveness of information systems from a product perspective, ignoring the service quality factor (Pitt et al., 1995). Subsequently, DeLone and McLean (2003) proposed a revised information systems success model to address the queries, criticisms, and extensions of the 1992 model in other studies, and added a “service quality” dimension in response to changes in the information systems environment, especially the emergence and rapid development of e-commerce. The usefulness of this updated information success model is evaluated by the e-business system, and the feedback relationship between system usage, user satisfaction, and net benefits is used to estimate the information effectiveness, which is considered as the success indicator of the information processing system.

### Relationship Quality

Relationship quality is a critical issue that is often discussed and studied in relationship marketing. Crosby et al. (1990) define relationship quality as a comprehensive assessment of the strength of the relationship between buyer and seller that meets both their needs and expectations. These needs and expectations are based on the past successes and failures of both buyers and sellers, and good relationship quality can re-duce service uncertainty. Although relationship quality has since been summarized as the treatment of intangible values, it does not embody the true meaning of relationship quality. However, as the research progresses, based on the study of the relationship between relationship quality and relationship benefits and their derivative behaviors, it is concluded that relationship quality is an appropriate level of satisfying the needs of the customer relationship. Based on the synthesis of current studies, relationship quality is defined as a high-level structure that contains various positive relationship outcomes, reflecting the influence of the relationship and the level of satisfaction of the relationship's needs and expectations.

Further, Hennig-Thurau and Klee (1997) demonstrate that relationship quality and product quality share the same concept and can both be considered as the degree to which consumer needs are met. In summary, relationship quality is a high-level concept that combines multiple positive relationship outcomes and reflects the overall strength of a relationship while meeting expectations and needs (Smith, 1998).

In recent years, the rapid development of the relevant research on the relationship between information system users and organizations. Among them, relationship quality is seen as an important mediating factor in inter-firm relationships (Chen, 2012; Chen et al., 2013b, 2014; Masri et al., 2020). Since there are many factors involved in the quality of corporate relationships, it is especially important to have multiple points of contact and key relationships between the two sides of the transaction in the process of developing closer relationships with customers. Although there is still debate in the past literature on how to measure relationship quality metrics, the current literature discusses at least two metrics in general, namely customer satisfaction and trust (Lin and Ding, 2009; Lin et al., 2011; Chen, 2012; Chen et al., 2013b, 2014; Masri et al., 2020). Therefore, this study uses two indicators of user satisfaction and trust in the cloud financial accounting system to measure the attitude of customers toward the financial accounting system, that is, the extent to which users perceive the quality of the relationship with the cloud financial accounting system. The two dimensions of satisfaction and trust in the relationship quality are described below.

#### Satisfaction

Satisfaction refers to the degree to which customers feel happy or disappointed with the product expectations, that is, if the goods or services provided by the company can be greater than what customers expect, then customer satisfaction can be increased. After the implementation of an information system, enterprises, or organizations often use various methods to evaluate the benefits of the system in order to fully understand its use. However, there are many factors used to estimate the performance of system implementation. In recent years, in the field of information system usage, the most common factors used to measure system implementation results include user satisfaction, system usage, user involvement, user acceptance, etc. Among them, user satisfaction is the most widely accepted and used factor (Hennig-Thurau and Klee, 1997; DeLone and McLean, 2003; Woodroof and Burg, 2003; Liao et al., 2007; Chen et al., 2009, 2013a; Chen and Lin, 2019).

#### Trust

In any relationship, trust is inevitably an important part of the relationship. Trust between two individuals is what makes a relationship sustainable and long-lasting. Trust is a necessary factor for building forceful market share and customer relationships (Urban et al., 2000). Before gaining the loyalty of customers, it is necessary to gain their trust (Reichheld and Schefter, 2000). The first field of research on trust is psychology, where psychologists investigate whether trust has an impact on interpersonal relationships. Trust is a concept often used in relationship marketing (Dwyer et al., 1987). Morgan and Hunt (1994) define trust as when a party to a transaction has confidence in the reliability and integrity of its counterparty, while Moorman et al. (1993) define trust as the willingness to rely on a confident counterparty in an exchange relationship and to have confidence in it, both of which mention the importance of the concept of confidence; therefore, in the transaction process, trust is generated because the buyer has confidence in the counterparty, which leads to the completion of the transaction. Trust is an important medium of connection between two individuals, and its existence can reduce the risk of mutual cooperation and ensure future benefits (Chow and Holden, 1997). Moorman et al. (1993) defined trust as the willingness to rely on a partner one trusts.

### Continuance Intention

The adoption of information systems has been one of the most common measures of information system success, especially as a behavioral proxy for information system effectiveness. In the literature related to information system behavior, ECM is often used to assess and measure consumer satisfaction with a product or service and as an infrastructure for post-purchase behavior (Bhattacherjee, 2001). In recent years, many different studies have attempted to investigate the continuous use of information systems or evaluate the performance of in-formation system use in different contexts, including E-learning (Lin, 2011, 2012), mobile banking (Chen, 2012; Zhou, 2013), online shopping (Al-Maghrabi and Dennis, 2011; Lu et al., 2017), educational services and knowledge sharing (Fang and Chiu, 2010; Cheng et al., 2015), adoption of e-books (Chen et al., 2018), adoption of wireless technologies and mobile networks (Chen et al., 2013a), adoption of social networking sites (Kim, 2011; Chen et al., 2012; Chen and Lin, 2019), and Enterprise Mobile Applications, among others. Therefore, in this study, the relationship between satisfaction of using cloud financial accounting system and continuous use intention was included in this research model.

Prior studies applied continuance intention as dependent variables because an effective and efficient quality improvement for continuance intention requires the understanding of specific quality dimensions that considerably influence continuance intention because service quality has multiple dimensions (Park et al., 2012; Kim et al., 2019). Therefore, this research evaluates the enhancement of an influential quality dimension improves continuance intention more effectively and efficiently than enhancing a less influential one from information system success perspective.

## Research Methodology

### Research Hypotheses Development

In the research field of information systems and information technologies, information quality, system quality, and service quality are often considered as the result of the use of a system. Thus, information quality can be considered as the ability of a financial accounting system to handle information in terms of accuracy, timeliness, relevance, completeness, and understandability (DeLone and McLean, 2004), while system quality is the ability of a system to be reliable, responsive, and flexible to maintain the operation of a cloud financial accounting system (Seddon, 1997); and service quality represents the overall service quality is the overall ability of a financial accounting system to provide good service (Pitt et al., 1995). According to DeLone and McLean (2003), information quality, system quality, and service quality have a positive impact on user satisfaction. Melchor and Julián (2008) followed the IS success model to measure decision information systems and found that information quality and system quality were influential factors in decision satisfaction. In addition, Negash et al. (2003) found that information quality and system quality had a positive effect on user satisfaction, but service quality didn't provide the significant effect on user satisfaction. Lee et al. (2007) found the direct effect of information quality and service quality on trust in a sample of 233 SMEs with ASP service experience, and Lee and Chung (2009) confirmed the significant effect of information quality and system quality on trust in an empirical study of mobile payment.

On the other hand, the information system model of DeLone and McLean (2003) is one of the most distinguished in the IS field (Petter et al., 2008). Prior research also indicated the information system model of DeLone and McLean (2003) can be used to evaluate accounting information system success (Al-Hattami, 2021). In this vein, cloud financial information system can be counted as information system, where information system quality is an important aspect of accounting information system. Through the above discussion, this study establishes the following hypotheses:

H1: Information quality has the positive impact on satisfaction.
H2: Information quality has the positive impact on trust.
H3: System quality has the positive impact on satisfaction.
H4: System quality has the positive impact on trust.
H5: Service quality has the positive impact on satisfaction.
H6: Service quality has the positive impact on trust.

Bhattacherjee (2001), in a study on the intention of continuous use of information systems, states that the satisfaction level that results from actual use will determine whether the user continues to use the system or not. Through the empirical results of ECM, we can find a link between satisfaction and continuance intention (Thong et al., 2006; Lee, 2010; Chen et al., 2013a; Li et al., 2019; Ruangkanjanases et al., 2020). In addition, previous related studies point to a positive relationship between satisfaction and trust (Flavián et al., 2006; Sanchez-Franco et al., 2009; He et al., 2012; Moreira and Silva, 2015). Therefore, through the above discussion, we deduced that H7 and H8 of this study:

H7: Satisfaction has the positive impact on trust.
H8: Satisfaction has the positive impact on continuance intention.

Chen et al. (2014) showed that users' trust level in adopting information systems positively influences the intention to continue using them. Previous scholars have also demonstrated in mobile payment service studies that persistent usage intention is significantly influenced by user trust (Zhou, 2013; Cao et al., 2018; Handarkho, 2020). Through the above empirical results, the H9 of this paper is as follows:

H9: Trust has the positive impact on continuance intention.

**Figure 2:**
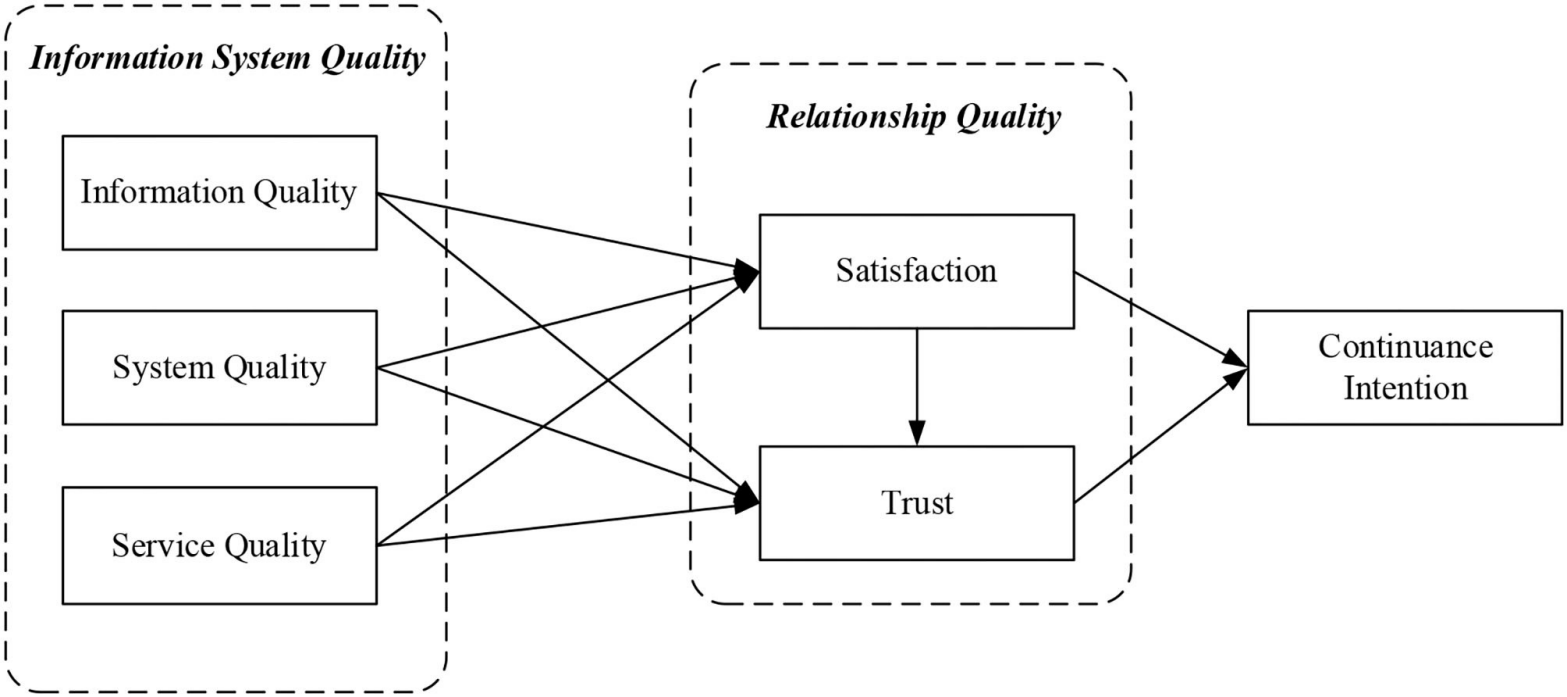
Research framework.

According to the discussion of hypotheses development, we proposed our research model and summary of research hypotheses (as shown in Figure 2 and Table 1). This study was conducted by three stages to develop the measurement items. First, the questions in this study were designed with reference to the relevant literature and were revised by three experts in the field and a professional consultant with knowledge of cloud financial accounting systems after the design was completed. Second, the structure and content of the questionnaire were designed based on domestic and foreign literature, re-search frameworks and their operational definitions, and the Likert seven-point scale was used to explore the information system measurement structure from the perspective of system users to measure the attitude and current situation of using cloud financial ac-counting systems. The questionnaire design consists of six main components. Information system quality is primarily a measure of what users of a system consider to be a successful information system. The factors analyzed by Lee et al. (2007) for system, information, and service quality dimensions were used as benchmarks to investigate the impact of the “information system measurement dimensions” of system quality, information quality, and service quality on satisfaction and trust. Satisfaction measurement items are mainly used to measure system users' satisfaction with using the cloud financial accounting system refers to the views of scholars such as Bhattacherjee (2001) and Limayem et al. (2007). The measurement items of trust and intention to continue using the system are mainly from Chen et al. (2013b). Third, this study was conducted in China to collect data and questionnaires from instructors and finance staffs who had used the cloud financial accounting system for reimbursement in the past. Five universities in China, having implemented cloud financial accounting systems accessible to all faculty members, agreed to participate voluntarily in the research. The subjects were those who owned the usage experiences of cloud financial accounting information systems 2 years at least, rather than those without usage experience, to ensure the external validity of this research. The investigation was issued using an online instrument in the spring and fall semesters of 2020. A total of 326 questionnaires were collected, and 289 valid respondents were returned after deducting 37 invalid questionnaires.

**Table 1:** Research hypotheses.

**Research hypothesis description**	**Direction**	**Source**
H1: Information quality has the positive impact on satisfaction	INQ → SAN	Lee et al., 2007; Fang et al., 2011; Aparicio et al., 2017
H2: Information quality has the positive impact on trust	INQ → TR	Lee et al., 2007; Zhou, 2013
H3: System quality has the positive impact on satisfaction	SYQ → SAN	Lee et al., 2007; Fang et al., 2011; Aparicio et al., 2017
H4: System quality has the positive impact on trust	SYQ → TR	Lee et al., 2007; Zhou, 2013
H5: Service quality has the positive impact on satisfaction	SERVQ → SAN	Lee et al., 2007; Fang et al., 2011; Aparicio et al., 2017
H6: Service quality has the positive impact on trust	SERVQ → TR	Lee et al., 2007; Zhou, 2013
H7: Satisfaction has the positive impact on trust	SAN → TR	Flavián et al., 2006; He et al., 2012
H8: Satisfaction has the positive impact on continuance intention	SAN → CINT	Bhattacherjee, 2001; Hsu et al., 2006; Liao et al., 2007; Chen et al., 2014; Ma et al., 2019
H9: Trust has the positive impact on continuance intention	TR → CINT	Zhou, 2013; Chen et al., 2014; Cao et al., 2018; Handarkho, 2020

*INQ, information quality; SYQ, service quality; SERVQ, service quality; SAN, satisfaction; TR, trust; CINT, continuance intention*.

## Empirical Data Analysis Results

In this study, Partial Least Square (PLS) was applied to evaluate our proposed model and SmartPLS 3.3.3 was used as the analysis tool (Ringle et al., 2015). Fornell and Bookstein (1982) showed that PLS is a highly suitable method for dealing with complex causal relationships. Partial Least Square is highly practical and superior to the general linear structural relationship model analysis techniques and is more lenient on the assumption of multivariate normal distribution, and sample size of the variables that must be met (Hair et al., 2016; Lin et al., 2020). In order to find the stability of each variable estimation, a bootstrap resampling procedure was used with 5,000 cycles (Hair et al., 2012). Therefore, PLS was used to analyze hypotheses 1–9 and to examine the effect of mediation.

### Outer Model Estimation

This study assessed the construct validity of the selected measures in relation to the constructs, and the validation factor analysis will help to further examine the convergent validity and discriminant validity of the scales. Before estimating construct validity, this study performed Harman's single-factor test to detect the potential influence from the estimation of exploratory factor analysis (EFA) with all the variables produces eigenvalues whether the first factor accounts for higher than 50% of the variance among variables (Podsakoff and Organ, 1986; Podsakoff et al., 2003). The EFA test of our data indicated the first factor accounted for 25% of the total variance. The results also showed that each principal component explained similar amounts of the total variance of 69.1%, ranging from 10.5 to 19.2%. This result revealed that our data did not suffer seriously from common method bias. In this study, Cronbach's alpha, composite reliability (CR), rho A, and average variance extracted (AVE) were used to assess the internal consistency of the constructs. According to Fornell and Bookstein (1982) and Fornell and Larcker (1981), the CR and AVE values should be higher than 0.60 and 0.50. In the present study, the CR values ranged from 0.84 to 0.93, and the AVE values ranged from 0.64 to 0.82 (as shown in Table 2). In addition, both Cronbach Alpha and rho A are higher than the previous scholars' requirements for internal consistency (Nunnally and Bernstein, 1994; Dijkstra and Henseler, 2015). From the above results, all four indicators are higher than the above criteria, indicating a high level of internal consistency in the measurement of the dimensions about this research.

**Table 2:** Convergent validity.

**Construct**	**Cronbach's alpha**	**rho_A**	**Composite reliability**	**Average variance extracted (AVE)**
Information quality	0.732	0.773	0.844	0.644
System quality	0.861	0.862	0.916	0.783
Service quality	0.832	0.854	0.887	0.664
Satisfaction	0.887	0.893	0.930	0.817
Trust	0.717	0.718	0.841	0.639
Continuance intention	0.805	0.814	0.885	0.720

The purpose of the discriminant validity analysis was to verify whether there were significant differences between the two different constructs. In this study, three methods were used to verify the discriminant validity. First, we checked whether the correlation coefficients between all potential constructs were significantly smaller than 1. As the results of the analysis in Table 3 show, none of the confidence intervals (correlation ± two standard errors) contained 1, indicating that they had discriminant validity (Bagozzi and Yi, 1988). Secondly, according to Fornell and Larcker (1981) and Hair et al. (2013), the diagonal element is the square root of the AVE for each construct and the criterion of discriminant validity is satisfied if any diagonal element is larger than the horizontal and vertical elements of the lower triangular matrix (i.e., the correlation coefficient between two constructs) (as shown is Table 4). Third, as shown in Table 5, the indicator loadings of each item on its own construct (in bold) are larger than the cross-loadings formed by the items and the other construct. This result indicates that there is acceptable with the effect of discriminant validity of the questions used in the two constructs of this study (Chin, 1998; Hair et al., 2016).

**Table 3:** Confidence intervals of correlations.

**Pairs of correlation**	**Correlation**	**Lower bound of CI (2.50%)**	**Upper bound of CI 97.50%**
(INQ, SYQ)	0.297	0.189	0.411
(INQ, SERVQ)	0.260	0.163	0.364
(INQ, SAN)	0.347	0.26	0.443
(INQ, TR)	0.595	0.521	0.67
(INQ, CINT)	0.501	0.426	0.581
(SYQ, SERVQ)	0.321	0.208	0.431
(SYQ, SAN)	0.422	0.308	0.534
(SYQ, TR)	0.493	0.388	0.589
(SYQ, CINT)	0.370	0.251	0.484
(SERVQ, TR)	0.498	0.411	0.585
(SERVQ, SAN)	0.531	0.445	0.613
(SERVQ, CINT)	0.499	0.409	0.586
(SAN, TR)	0.714	0.65	0.773
(SAN, CINT)	0.793	0.739	0.841
(TR, CINT)	0.763	0.705	0.818

*CI, confidence interval; INQ, information quality; SYQ, service quality; SERVQ, service quality; SAN, satisfaction; TR, trust; CINT, continuance intention*.

**Table 4:** Discriminant validity.

**Construct**	**INQ**	**SYQ**	**SERVQ**	**SAN**	**TR**	**CINT**
Information quality	***0.803***					
System quality	0.297	***0.885***				
Service quality	0.260	0.321	***0.815***			
Satisfaction	0.347	0.422	0.531	***0.904***		
Trust	0.595	0.493	0.498	0.714	***0.799***	
Continuance intention	0.501	0.370	0.499	0.793	0.763	***0.848***

*INQ, information quality; SYQ, service quality; SERVQ, service quality; SAN, satisfaction; TR, trust; CINT, continuance intention*.

**Table 5:** Factor loadings and cross loadings.

**Indictor**	**Information quality**	**System quality**	**Service quality**	**Satisfaction**	**Trust**	**Continuance intention**
INQ1	***0.707***	0.120	0.082	0.132	0.321	0.268
INQ2	***0.875***	0.344	0.213	0.303	0.532	0.459
INQ3	***0.816***	0.211	0.281	0.346	0.530	0.436
SYQ1	0.273	***0.903***	0.276	0.401	0.438	0.310
SYQ2	0.239	***0.895***	0.300	0.378	0.401	0.359
SYQ3	0.276	***0.857***	0.277	0.340	0.469	0.314
SERVQ1	0.230	0.294	***0.846***	0.558	0.440	0.484
SERVQ2	0.182	0.304	***0.854***	0.451	0.415	0.420
SERVQ3	0.212	0.262	***0.848***	0.399	0.404	0.391
SERVQ4	0.232	0.161	***0.703***	0.268	0.358	0.301
SAN1	0.285	0.328	0.490	***0.875***	0.633	0.690
SAN2	0.356	0.387	0.547	***0.942***	0.698	0.752
SAN3	0.297	0.431	0.394	***0.893***	0.600	0.706
TR1	0.398	0.341	0.493	0.604	***0.807***	0.543
TR2	0.519	0.554	0.375	0.589	***0.769***	0.554
TR3	0.504	0.289	0.335	0.523	***0.821***	0.723
CINT1	0.374	0.319	0.496	0.722	0.576	***0.825***
CINT2	0.359	0.339	0.343	0.589	0.602	***0.823***
CINT3	0.529	0.290	0.425	0.701	0.754	***0.895***

### Inner Model Analysis

**Figure 3:**
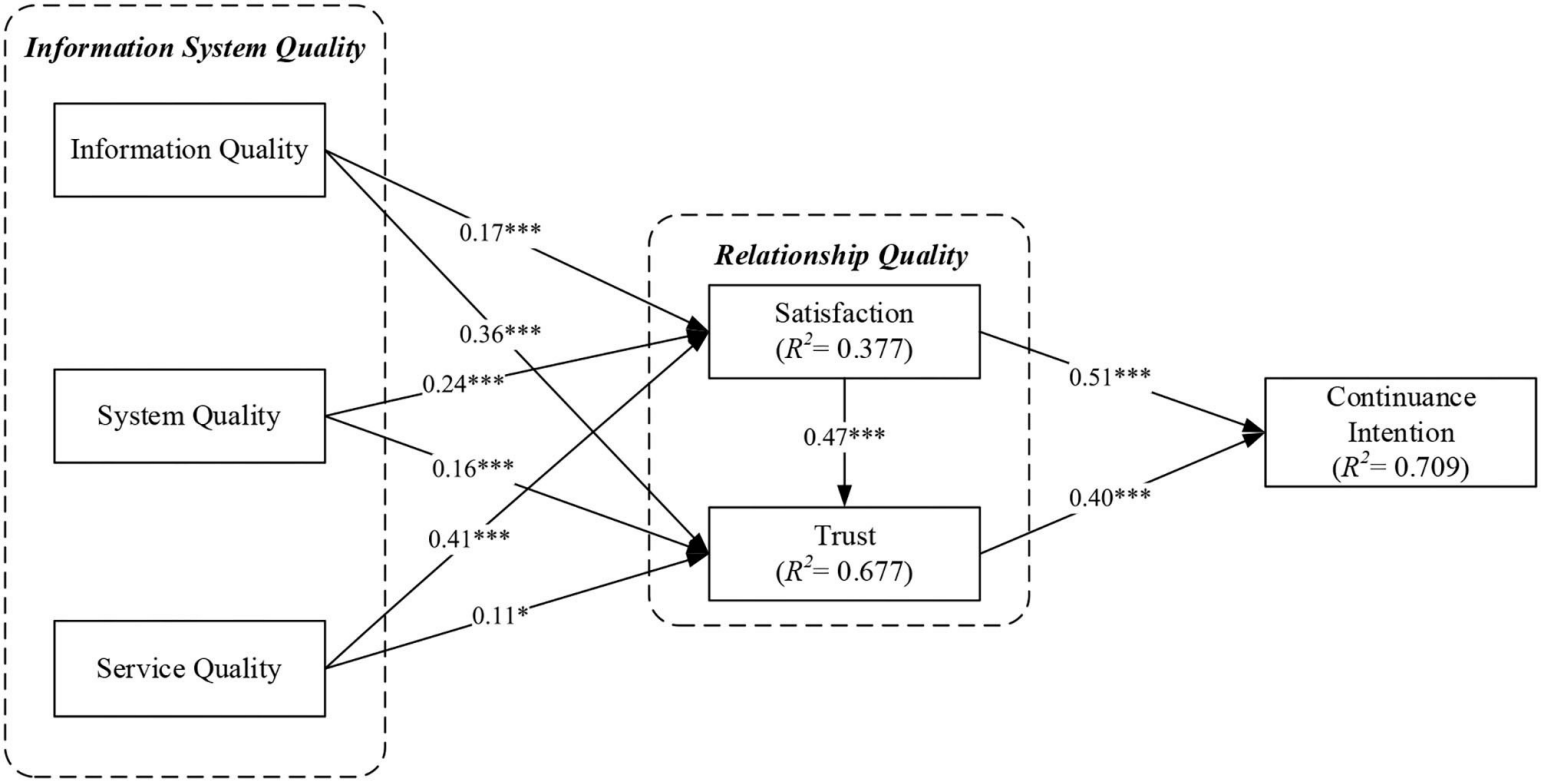
Inner model path coefficients and significant level. ^*^p-value < 0.05; ^***^p-value < 0.001.

This section focuses on the analysis of the internal model, using SmartPLS 3.3.3 software to analyze and validate the causal relationships between the potential variables of the structural model and to investigate the explanatory power of the model using *R*^2^ judgments (Pavlou and Fygenson, 2006). The *R*^2^-value is the percentage of variance explained by exogenous variables to endogenous dimensions and demonstrates the predictive power of our proposed model, ranging from 0 to 1. The larger the *R*^2^-value, the better the explanatory power of the model. According to the nine hypotheses proposed in the research framework and the results of the overall pattern relationship path check presented in Figure 3 (statistically significant as solid lines), all nine path relationships were significant. The results of the hypothesis validation are organized as shown in Table 6. The results of this study revealed that the satisfaction level of cloud financial accounting system adoption was positively and significantly affected by the quality of information quality, system quality, and service quality, and the explanatory power of these three important dimensions on the variation of satisfaction level was 37.7%. In addition to the three dimensions of information system quality, user satisfaction also has a positive and significant impact on trust, resulting in 67.7% of variance explanation power. Finally, the positive effect of information system quality through satisfaction and trust on the continuous use intention of the cloud financial accounting system resulted in an explanatory power of 70.9% of the variance on continuance intention toward cloud financial information system. The results showed that the explanatory power of this model is quite good and meets the criteria suggested by Cohen (1988).

**Table 6:** Inner model analysis result.

**Path direction**	**Standardized path coefficient**	**Standard deviation (STDEV)**	***T*-statistics**	***P*-values**
INQ → SAN	0.170^***^	0.046	3.677	0.000
INQ → TR	0.359^***^	0.047	7.562	0.000
SYQ → SAN	0.240^***^	0.054	4.485	0.000
SYQ → TR	0.155^***^	0.037	4.187	0.000
SERVQ → SAN	0.410^***^	0.050	8.271	0.000
SERVQ → TR	0.107^*^	0.043	2.515	0.012
SAN → TR	0.467^***^	0.046	10.168	0.000
SAN → CINT	0.506^***^	0.064	7.894	0.000
TR → CINT	0.402^***^	0.066	6.052	0.000

*INQ, information quality; SYQ, service quality; SERVQ, service quality; SAN, satisfaction; TR, trust; CINT, continuance intention*.

* *p < 0.05*;

*** *p < 0.001*.

Due to the small sample size in this study, scholars suggest using bootstrap analysis to examine the effects of mediation (MacKinnon et al., 2002; Shrout and Bolger, 2002). Bootstrap is a widely used method for repeatedly extracting a large number of samples when the source of the data is unknown (no independent observations can be established) and the number of data is limited, providing a more accurate approximation than the commonly used limit approximation. This can be handled even when the parent body is not suitable to be described by a normal assignment (MacKinnon et al., 2002; Shrout and Bolger, 2002). Accordingly, in this study, we used this method to verify the effect of in-formation system quality through relational quality on the intermediation of ongoing use intentions by replicating 5,000 samples and obtaining the confidence interval for each parameter in this model, and then determining its significance based on the above principles. According to Table 7, the 95% confidence interval for all intermediary paths was significantly larger than 0 ([0.141, 0.491]), suggesting that three different information system qualities play a full intermediary role between ongoing adoption of cloud financial ac-counting systems and relationship quality (Shrout and Bolger, 2002).

**Table 7:** Mediation effect result.

**Mediation path**	**Effect size**	**95% confidence interval**	***T*-statistics**
INQ → SAN → TR	0.079^**^	(0.039, 0.128)	3.409
INQ → SAN → CINT	0.086^**^	(0.040, 0.148)	3.103
INQ → TR → CINT	0.144^***^	(0.079, 0.021)	3.964
SYQ → SAN → TR	0.112^***^	(0.066, 0.166)	4.390
SYQ → SAN → CINT	0.122^***^	(0.075, 0.181)	4.492
SYQ → TR → CINT	0.062^***^	(0.038, 0.094)	4.312
SERVQ → SAN → TR	0.191^***^	(0.134, 0.256)	6.095
SERVQ → SAN → CINT	0.207^***^	(0.140, 0.288)	5.458
SERVQ → TR → CINT	0.043^*^	(0.011, 0.086)	2.279
SAN → TR → CINT	0.188^***^	(0.122, 0.259)	5.324
INQ → SAN → TR → CINT	0.032^**^	(0.016, 0.056)	3.201
SYQ → SAN → TR → CINT	0.045^**^	(0.022, 0.077)	3.180
SERVQ → SAN → TR → CINT	0.077^***^	(0.047, 0.116)	4.393

*INQ, information quality; SYQ, service quality; SERVQ, service quality; SAN, satisfaction; TR, trust; CINT, continuance intention*.

* *p < 0.05*;

** *p < 0.01*;

*** *p < 0.001*.

## Discussion

The results obtained from the above analysis showed that all the hypotheses of this study were valid. Based on the results of the analysis, the findings of this study are summarized and discussed as follows. First, the information quality of the accounting system had a positive and significant effect on satisfaction and trust (IQ → SAN: *β* = 0.170, *t* = 3.677, *p* < 0.001; INQ → TR: *β* = 0.359, *t* = 7.562, *p* < 0.001). The results of this study are consistent with previous findings (Lee et al., 2007; Rahi and Ghani, 2019). In addition, in this study, it was found that information quality has a significantly higher impact on trust than satisfaction. It is also found from previous studies that data security is a well-known and challenging issue (Dawson and Thomson, 2018; Kang et al., 2019). Therefore, the risk arising from information quality is a key factor in choosing a cloud financial and accounting system (Demirkan et al., 2020). Moreover, users face high operational and business risks in selecting and maintaining the services of cloud financial accounting systems. The development of information system and the provision of information quality will facilitate the rapid collection, storage, and exchange of knowledge with the improvement of communication and cooperation in organizations (Gold et al., 2001; Hossain and Wigand, 2004; Appelbaum et al., 2017). Therefore, the higher the quality of information in the financial and accounting system, the more positive the impact on trust building.

Second, through user satisfaction and trust, system quality will have a positive and significant effect on the intention of continued use of the cloud financial accounting system (SYQ → SAN: *β* 0.240, *t* = 4.485, *p* < 0.001; SYQ → TR: *β* = 0.155, *t* = 4.187, *p* < 0.001). With the advancement of information technology, the demands of users are diversifying. The service providers of financial/accounting systems should maintain close contact with users, design methods, and procedures to track their demands, and respond to customers' questions quickly and correctly (Taipaleenmäki and Ikäheimo, 2013). In addition, the cloud service providers should pay attention to the customer's usage of the financial/accounting system and the perception of the company's service, regularly consult the customer to see if their needs have been met, and immediately seek improvement and propose solutions to address dissatisfaction (Bhattacherjee and Park, 2014).

Third, this study found that the influence and importance of service quality components on satisfaction was significantly higher than the influence on trust (SERVQ → SAN: *β* = 0.410, *t* = 8.271, *p* < 0.001; SERVQ → TR: *β* = 0.107, *t* = 2.515, *p* < 0.05). Bitner et al. (2000) mentioned in their study that the infusion of technology enhances the flexibility and efficiency of service delivery. Therefore, if the user sees the information system as an aid rather than a complete replacement for the human interface, the aid of the information system can indeed make the interaction with the service provider more convenient, thus increasing the user's perceived satisfaction with the service.

## Conclusion

This research has important practical implications that could help the system developers and solution providers to ameliorate and improve user's continuance intention to use cloud financial accounting system via satisfaction and trust. The integration of information system success model and relationship quality showed significant impact on continuance intention to adopt cloud financial accounting system. The findings also imply that system developers and solution providers seeking satisfaction and trust of users should focus on information quality, system quality service quality to improve users' continuance intention to adopt cloud financial accounting system.

This research focuses on the D&M information system success model as the theoretical basis to investigate the continuous use behavior and intention of cloud financial ac-counting systems. Trust has recently received much attention in the literature of knowledge management systems and has been conceptually explored in the literature from many different perspectives. The conceptual literature has explored many different perspectives. For instance, Resource Based Theory (RBT), Transaction Cost Economics, Social Network For example, RBT, Transaction Cost Economics, Social Network Theory, etc. Regardless of the views of the foundations, the issue of trust is seen as an important factor in the success of information systems (Chowdhury, 2005). Unfortunately, however, empirical studies are still lacking. This study incorporates trust into the D&M IS Success Model as a systematic measure, which not only corroborates and echoes previous conceptual theories with empirical evidence, It also shows that user psychological factors are much more important than information system factors. The D&M IS Success Model has been included as a measure of the service quality of information systems, but it fails to identify the interaction between system quality, information quality, and service quality. This study uses relational quality to interpret the causal relationship between information system quality and continuous use intention and supports this inference with empirical results. In other words, if users can perceive that the service quality of IS has improved, they will agree that the quality of system hardware, software, data, and reports have improved (Li et al., 2021). Therefore, in order to successfully implement the cloud financial accounting system, to enhance the performance and service quality should be from the first priority information department.

This research denoted effort to be rigorous and thorough in all aspects of this study, with the hope of providing a referable and trustworthy result. However, there was still room for improvement in the research due to time and manpower constraints. First, PLS required that the sample size must be larger than the total number of samples collected, preferably 10 times larger (Hair et al., 2016). Although the sample size of this study was already higher than this standard, this study was mainly based on the use of financial and accounting systems by instructors in the universities of China, which still limited the generalization and external validity of the findings and theoretical models. It was suggested that subsequent researchers can extend the study to other organizations and enterprises with other types of cloud system services to conduct cross-country or cross-culture comparative studies of cloud system adoption behaviors. Second, this study used quantitative research to analyze and investigate, and subsequent research can conduct qualitative research on related issues and would develop a new research framework to deeply explore the antecedents and consequences of information system service quality. Finally, this study used the investigation to collect respondents, therefore, subsequent studies can increase the sample size and collect secondary data for quantitative research to test the validity of the theoretical framework of this study.

## Data Availability Statement

The raw data supporting the conclusions of this article will be made available by the authors, without undue reservation.

## Author Contributions

YL and JW: conceptualization, writing—original draft preparation, and writing—review and editing. YL: methodology, formal analysis, and investigation. JW: visualization. All authors have read and agreed to the published version of the manuscript.

## Conflict of Interest

The authors declare that the research was conducted in the absence of any commercial or financial relationships that could be construed as a potential conflict of interest.

## Publisher's Note

All claims expressed in this article are solely those of the authors and do not necessarily represent those of their affiliated organizations, or those of the publisher, the editors and the reviewers. Any product that may be evaluated in this article, or claim that may be made by its manufacturer, is not guaranteed or endorsed by the publisher.
